# A Pilot Study to Assess Food Safety and Potential Cholesterol-Lowering Efficacy of *Antrodia cinnamomea* Solid-State Cultivated Mycelium in Healthy Adults

**DOI:** 10.1155/2020/5865764

**Published:** 2020-04-09

**Authors:** Wan-Jing Chen, Fung-Wei Chang

**Affiliations:** ^1^Ph. D. Program in Nutrition and Food Science, Fu Jen Catholic University, No. 510, Zhongzheng Rd., Xinzhuang Dist., New Taipei City 24205, Taiwan; ^2^Attending Physician of Department of Clinical Research Center, National Defence Medical Center, Tri-Service General Hospital, No. 325, Sec. 2, Chenggong Rd., Neihu District, Taipei City 11490, Taiwan; ^3^Director of Department of Obstetrics and Gynecology, National Defence Medical Center, Tri-Service General Hospital, No. 325, Sec. 2, Chenggong Rd., Neihu District, Taipei City 11490, Taiwan

## Abstract

*Antrodia cinnamomea* is a Taiwanese medicinal mushroom with multiple pharmacological activities. *Antrodia cinnamomea* solid-state cultivated mycelium (LAC) exerts health-related effects in animal and cell models, but clinical data is limited. This study aimed to determine the safety and effects of LAC on human physiological functions. In an open-label, single-arm study, 32 healthy men and women ingested LAC capsules for three months. The subjects were monitored during the study and one month after the study end-point. LAC consumption did not significantly change fasting blood glucose, blood pressure, and triglyceride levels or liver and renal function indices. No adverse events occurred during the trial. Moreover, a significant change from baseline in total cholesterol levels was observed; men and women had decreases of 5.7% and 5.3%, respectively. Based on these, the ingestion of LAC-capsule has a considerable degree of safety and has the potential to reduce total cholesterol in healthy adults.

## 1. Introduction

*Antrodia cinnamomea* (syn. *Taiwanofungus camphoratus*, *Antrodia camphorata*) has long been used in traditional Chinese medicine to protect against various health issues such as liver disease, drug and food intoxication, diarrhea, and cancer [1, 2]. Its medicinal properties are due to the fact that this fungal species contains more than 78 bioactive compounds, many of which have been identified, including terpenoids, polysaccharides, benzenoids, lignans, nucleic acids, benzoquinone derivatives, steroids, and maleic/succinic acid derivatives [2, 3]. Among them, triterpenoids and polysaccharides have the most clinical application value, such as anticancer studies show that polysaccharides at 800 *μ*g/ml can significantly inhibit the viability of A549 cells by downregulating EGFR signal transduction [4]. Moreover, streptozotocin- (STZ-) induced diabetic animal models have found that triterpenoids in *Antrodia cinnamomea* could enhance the anti-inflammatory response, thereby inhibiting the diabetic chemokine (C-C motif) ligand 1 (CCL1) and thyroid peroxidase (TPO) expression [5]. However, the biologically bioactive compounds of *Antrodia cinnamomea* vary drastically depending on the culture method, extraction site, and type of product.

Recent studies have revealed that *Antrodia cinnamomea* mycelium extract has a variety of biological activities, such as hypolipidemic, antidiabetic [3, 6, 7], anticancer [8–10], anti-nflammatory [11, 12], liver protection [13–15], and immunomodulatory effects [16, 17]. The toxicology and effects of solid-state cultivated mycelium powder of *Antrodia cinnamomea* on liver function and immune regulation have been investigated in cell and animal models in the health food industry; [18–20] however, clinical data is still limited. To verify the food safety and potential efficacy of *Antrodia cinnamomea* solid-state cultivated mycelium, we conducted a nonrandomized, open-label, single-arm study in healthy adults to examine changes in blood biochemical parameters before and after *Antrodia cinnamomea* consumption.

## 2. Materials and Methods

### 2.1. Study Participants

The research protocol for this study was approved by the Institutional Review Board of Tri-Service General Hospital (Taipei, Taiwan; IRB No. 2-107-05-158). All participants provided informed consent before their enrollment in the study. The inclusion criteria for this clinical trial include (1) the subject's age is ≥ 20 years, (2) the subject is judged to be a healthy subject at the physical and psychological levels by nonclinical significance through interviews with the trial physician and inspection of the disease history and clinical examination results, (3) the subject should also have normal blood, biochemical, and urine clinical test values judged by the test physician, and (4) subjects with nonclinical significant signs or symptoms may still be recruited if judged by the trial physician that it will not affect the results of the study.

Eligible participants were men or women between 20 and 60 years of age, and the exclusion criteria were as follows: (1) subjects had severe uncontrollable clinical diseases, such as heart, liver, kidney, and infectious diseases. (2) Any subject who is diagnosed with a disorder such as psychosocial disorder. (3) Based on the professional judgment of the trial physician, it is not recommended to include the subject for the safety consideration of the subject. (4) Subjects who have donated 250 to 500 mL of blood within two months. (5) A history of HIV, hepatitis B, or hepatitis C infection. (6) An allergic or intolerant reaction to any component of the test substance is known or suspected. (7) The subject has allergic conditions such as allergic asthma, rhinitis or atopic dermatitis and the test physician believes that this may endanger the subject's safety, interfere with the evaluation of the test, or affect the validity of the test results. (8) The subject has an autoimmune disease, such as systemic lupus erythematosus (SLE), Sjogren's syndrome, or rheumatoid arthritis. (9) The results of the pregnancy test were positive for the female subject before the trial began. (10) Female subjects are breastfeeding. (11) *Antrodia cinnamomea-* or *Ganoderma lucidum*-related products were used within one month before the test. (12) People who may need to take a proprietary or prescription drug a week before the test ends. Finally, 32 subjects met the criteria and agreed to participate in the entire clinical trial.

### 2.2. Study Design

An open-label, single-arm design was used and all procedures were in compliance with the CONSORT statement and the World Medical Association Code of Ethics. Each subject ingested two LAC-Capsules (containing 380 mg of *Antrodia cinnamomea* solid-state cultivated mycelium) twice a day (morning and evening) for three months. The LAC-Capsule contains 99% solid-state cultivated powder of *Antrodia cinnamomea* and 1% magnesium stearate and is manufactured by Taiwan Leader Biotech Corporation (Taipei, Taiwan).

A monitoring visit is conducted once a month during the trial period, and a follow-up monitoring visit is performed after the third month of the trial. Each monitoring visit is accompanied by physiology and blood examinations, blood biochemical analysis, and urine examination. All examinations were performed by the pathological examination department of the Tri-Service General Hospital.

### 2.3. Measurements

Physical examination includes temperature, heartbeat, and blood pressure. Blood examination includes Heme (Hb), WBC with classification count (WBC with differential), red blood cells (RBC), platelets, hematocrit (Hct), mean hematocrit Volume ratio (MCV), average red blood cell hemoglobin amount (MCH), and average red blood cell hemoglobin concentration (MCHC). Blood biochemical analysis includes alkaline phosphatase (Alk-P), alanine transaminase (ALT), aspartate aminotransferase (AST), albumin, blood urea nitrogen (BUN), alanine transferase (*γ*-GT), total bilirubin, total cholesterol (TC), triglyceride (TG), total protein, creatinine, fasting blood glucose, uric acid, ACTH, cortisol. Urine examination includes urine pH, white blood cells (WBC), red blood cells (RBC), urine protein (Protein), urine cylinder (Cast) test.

### 2.4. Statistical Analyses

Results are presented as mean ± standard deviation or median. Statistical analysis was performed by paired sample *t*-test and Pearson correlation using SPSS 22.0 software (SPSS Inc., Chicago, IL, USA). A *p* value of less than 0.01 (*p* < 0.01) was considered statistically significant.

## 3. Results

**Figure 1:**
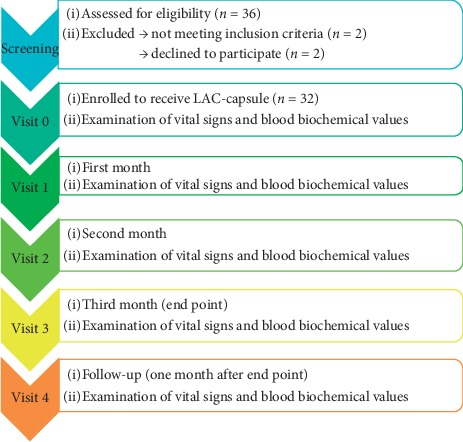
Flow diagram of study participation. ITT, intent-to-treat; PP, per-protocol.

This study recruited 36 healthy participants, and those who did meet trial inclusion criteria (*n* = (2) and discontinued participation due to personal reasons (*n* = (2) were excluded during the screening. A total of 32 subjects were finally included in the clinical study. Monitoring visits were conducted once a month, and follow-up monitoring was performed one month after the end-point (Figure 1). The demographic characteristics and age distribution at the time of initial screening are shown in Tables 1 and 2 Most of the subjects were between 20–40 years old (78%) (Table 2), were mildly overweight, and had normal heartbeat, blood pressure, and blood glucose (Table 1). Subjects took two solid culture mycelium capsules (LAC-capsule) in the morning and evening, with a compliance rate of 99.0% (data not shown). The composition of LAC-capsule contains solid-state cultivated powder of *Antrodia cinnamomea* (99%) and magnesium stearate (1%) as excipient (Table 3). The bioactive compounds of LAC-capsule include total triterpenes 44–66 mg/g, total polysaccharides 104–156 mg/g, and adenosine 0.6–0.9 mg/g.

### 3.1. Safety Assessment of LAC on Cardiovascular-Related Indicators in Healthy Adults

The values for cardiovascular related factors at baseline and end-point are shown in Table 4. After the subjects took LAC capsules for three months, the blood pressure, systolic blood pressure, and fasting blood glucose values at the end-point were not significantly different from the baseline. We observed a mild increase in triglyceride but still within the normal range, while total cholesterol significantly decreased by 5.1% (from 196.0 to 186.0 mg/dL, *p* < 0.01, 95% confidence interval (CI): −4.9%, −6.1%).

### 3.2. LAC Effectively Reduces Total Cholesterol Concentration in Healthy Adults

**Figure 2:**
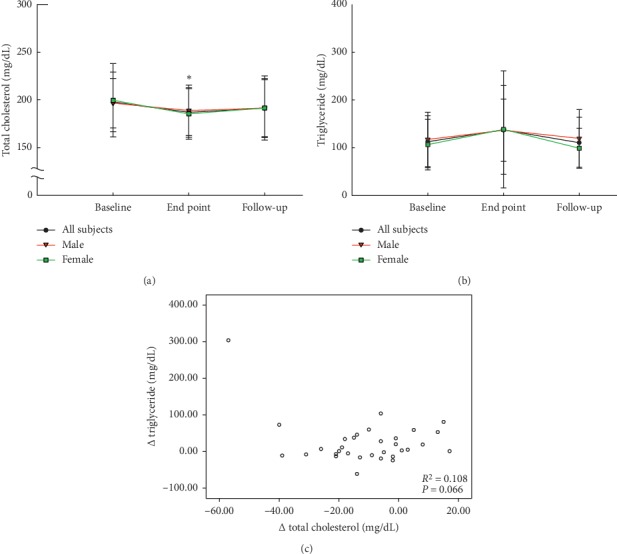
Changes and correlation in total cholesterol and triglyceride after the subjects took LAC-capsule for three months. The mean (a) total cholesterol and (b) triglyceride levels in male (*n* = 18) and female (*n* = 14) subjects were analyzed at baseline, end-point, and follow-up. (c) Correlation between changes in total cholesterol and triglycerides was examined. Data are expressed as mean ± SD. ^*∗*^Statistically significant (*p* < 0.01) when compared with baseline.

To further analyze the changes in total cholesterol and triglycerides of the subjects, a follow-up visit was conducted one month after the end-point of the trial to determine whether LAC-capsule exerts different effects between men and women (Figures 2(a), 2(b)). The results show that both male and female subjects had a significant reduction in plasma total cholesterol of 5.7% and 5.3%, respectively (*p* < 0.01), at the end-point compared to baseline, and a slight increase of 2.3–2.6% (191.4–191.6 mg/dL) in the follow-up assessment after discontinuation of LAC consumption (Figure 2(a)). In addition, gender does not affect the trend of triglyceride (Figure 2(b)). In general, the TG of male and female increased to 65.4 and 122.5 mg/dL at the end point, respectively, and decreased to 60.4 and 41.7 mg/dL after one month.

Next, we clarify whether there is a correlation between the two phenomena that LAC-capsule reduced cholesterol and mildly increased triglycerides.  Figure 2(c) indicated that there is no significant linear relationship between changes in total cholesterol and triglycerides (*p*=0.066, *R*^2^ = 0.108). This means that a decrease in total cholesterol is not significantly associated with a slight increase in triglycerides.

### 3.3. Safety Assessment of LAC on Liver and Renal Functions in Healthy Adults

Finally, we evaluated the effects of LAC-Capsule consumption on liver and kidney functions (Table 5). All subjects had normal blood biochemical values when screened for trial recruitment. After 12 weeks of intervention, the hepatobiliary indices of subjects, including AST, ALT, total bilirubin, ALP, and gamma GT were not significantly different from baseline, except for albumin, which decreased by 1.1% although the value was still within the normal range. There were no significant differences in the indicators of renal function including BUN, creatinine, and uric acid levels before and after intervention. Taken together, these results confirmed the safety of LAC-Capsule on liver and kidney functions.

## 4. Discussion

*Antrodia cinnamomea* may contain different bioactive compounds due to differences in culture (liquid, solid, or wood culture) and extraction methods and locations. The solid-state culture *Antrodia cinnamomea* mycelium powder (LAC) consumed as a health food in Taiwan contains two main bioactive compounds, triterpenoids (≥20.00 mg/g) and adenosine (≥0.20 mg/g), and its safety and specific and nonspecific immune regulatory functions have been confirmed by animal studies [18, 19]. This is the first study to evaluate the safety and efficacy of the solid-state culture mycelium of *Antrodia cinnamomea* on healthy humans.

Although we found that LAC did not affect blood pressure in healthy subjects, previous studies have revealed that patients with mild hypertension who were treated with *Antrodia cinnamomea* mycelium (including adenosine (1.18 mg/g), antrosterol (4.52 mg/g), and GABA (5.15 mg/g)) for two months showed reduced hypertension. This indicates that although the mycelium of *Antrodia cinnamomea* does not affect blood pressure in healthy adults, it may promote the health of patients with mild hypertension. [21] Similarly, although the results of the present study indicate that *Antrodia cinnamomea* mycelium has no significant effect on blood glucose and triglyceride levels in healthy subjects, it has been shown to be effective in improving obesity and hypertriglyceridemia in both animal and human trials [22–24].

During the entire clinical trial, the blood biochemical values of the subjects were regularly monitored every month. It was noted that some subjects had elevated triglycerides, but most of them remained within the normal range. A small number of subjects had high triglyceride values due to occasional changes in dietary habits, but they returned to normal during subsequent follow-up. Based on these, the doctor of the study site determined that there is no clinical significance (NCS) between increased triglyceride and using LAC-capsule. Next, we analyzed whether the decrease in cholesterol was related to the increase in triglycerides. The results of the Pearson correlation analysis showed that *R*^2^ = 0.108 and *p*=0.066 meaning that the changes of these two phenomena had no significant correlation in this study.

The present study only analyzes total cholesterol and does not do more blood lipid profiling. However, compared with other studies that *Antrodia cinnamomea* mycelium extract that contained antroquinonol significantly lowered LDL cholesterol levels in subjects with marginal and mildly elevated LDL cholesterol levels [25], this means that antroquinonol may play a role in the cholesterol-lowering effects of *Antrodia cinnamomea*.

One month after the study end-point, there was no significant difference (only 2.3–2.6% rebound) in total cholesterol levels compared with the end-point, indicating that even if LAC consumption were discontinued, the subjects retained the positive health promotion effects for at least 4 weeks. We also confirmed that LAC had no adverse effects on the kidney function of healthy subjects, and the change in albumin was also within the normal range, which is consistent with our toxicity test results in an animal model. [18, 19] The data shows that oral LAC has no adverse effects on liver and kidney function in adults.

LAC capsules were used as health food from 2009 to 2018 and were notified by the Taiwan Food and Drug Administration (TFDA) without any adverse events. Furthermore, no adverse events were observed during the clinical trial and subsequent follow-up, including diarrhea, constipation, headache, dizziness, nausea, and other physical discomforts. It is shown that the safety of *Antrodia cinnamomea* extract powder at this dose to the human body is relatively stable.

## 5. Conclusion

*Antrodia cinnamomea* has become a popular health food because of its liver protection, immune regulation, antioxidation, and anticancer effects, but its clinical application has not been explored and its effects on healthy adults are unknown. Our study confirms that *Antrodia cinnamomea* solid-state cultivated mycelium (LAC) has the potential to reduce marginal high total cholesterol with no adverse effects on liver and renal functions. In conclusion, we have confirmed our initial hypothesis that *Antrodia cinnamomea* solid-state cultivated mycelium powder is safe for human consumption and has health benefits. Our findings may provide a contribution of clinical value to the food or pharmaceutical industry.

## Figures and Tables

**Table 1:** Demographic characteristics of participants at screening (*n* = 36)^1^.

Variable	Value
Sex, male/female, *n*	21/15
Age, *y*	36.0 ± 7.84
Weight, kg	66.7 ± 12.35
BMI, kg/m^2^	24.2 ± 3.62
Heart rate, beats/min	77.9 ± 10.60
Systolic blood pressure, mm Hg	117.0 ± 16.05
Diastolic blood pressure, mm Hg	69.3 ± 10.91
Fasting blood glucose, mg/dL	93.2 ± 6.36

^1^All values included all subjects. ^*∗*^Mean ± SD (all such values for quantitative data).

**Table 2:** The age distribution of subjects (*n* = 32).

Age	Male (*n*)	Female (*n*)	Total (*n*)
≤20	0	0	0
21–30	6	4	10
31–40	8	7	15
41–50	4	2	6
51–60	0	1	1
≥61	0	0	0
Total (*n*)	18	14	32

**Table 3:** The composition of each LAC-Capsule offered to the subjects.

Test substance	LAC-Capsule
Package	460 mg/capsule
Capsule shell composition (80 mg)	Hydroxypropylmethyl cellulose
Ingredients (380 mg/capsule)	Solid-state cultivated powder of *Antrodia cinnamomea* (99%) Magnesium stearate (1%)
Bioactive compounds	Total triterpenoids: 44–66 mg/g Total polysaccharide: 104–156 mg/g Adenosine: 0.6–0.9 mg/g

**Table 4:** Cardiovascular-related factors at baseline and end of intervention and percentages of change from baseline^1^.

Variable	Baseline	End point	% of Δ
Systolic blood pressure, mm Hg	119.0 (111.4, 122.5)	115.5 (112.1, 119.3)	−2.9 (0.6, −2.7)
Diastolic blood pressure, mm Hg	70.5 (65.5, 73.0)	72.0 (67.6, 74.2)	2.1 (3.2, 1.6)
Fasting blood glucose, mg/dL	93.0 (91.0, 95.4)	94.5 (91.9, 97.5)	1.6 (1.0, 2.2)
Triglycerides, mg/dL	100.0 (93.6, 131.4)	128.0 (105.1, 169.5)	28.0 (12.3, 29.0)
Total cholesterol, mg/dL	196.0 (187.0, 208.8)	186.0 (177.9, 196.0)	−5.1 (−4.9, −6.1)^*∗*^

^1^All values are medians (95% CIs). Results are from the per-protocol sample. ^*∗*^Between-group differences in the cardiovascular factor were accessed by the paired sample *t*-test at *p* values < 0.01.

**Table 5:** Liver and renal function indices at baseline and end of intervention and percentages of change from baseline^1^.

Variable	Baseline	End point	% of Δ
AST, U/L	16.5 (16.2, 19.8)	16.0 (15.3, 19.3)	−3.0 (−5.5, −2.8)
ALT, U/L	15.0 (15.9, 25.1)	14.0 (13.8, 23.0)	−6.7 (−13.2, −8.3)
Total bilirubin, mg/dL	0.6 (0.6, 0.8)	0.6 (0.6, 0.8)	0.0 (−2.8, 0.5)
ALP, U/L	49.5 (47.3, 55.7)	51.0 (47.7, 55.0)	3.0 (0.7, −1.3)
Total protein, g/dL	7.3 (7.2, 7.4)	7.2 (7.1, 7.3)	−1.4 (−1.4, −1.5)
Albumin, g/dL	4.6 (4.6, 4.8)	4.6 (4.4, 4.6)	−1.1 (−2.9, −3.3)^*∗*^
Gamma GT, U/L	18.0 (16.1, 29.3)	15.0 (15.7, 24.7)	−16.7 (−2.3, −15.8)
BUN, mg/dL	13.0 (12.3, 14.9)	13.0 (12.0, 14.9)	0.0 (−2.1, 0.5)
Creatinine, mg/dL	0.8 (0.7, 0.8)	0.8 (0.7, 0.8)	0.0 (−1.2, −2.0)
Uric acid, mg/dL	5.4 (4.9, 5.9)	5.6 (4.9, 5.9)	4.7 (−0.4, 0.4)

^1^All values are medians (95% CIs). Results are from the per-protocol sample. ^*∗*^Between-group differences in the liver and renal factor were accessed by the paired sample *t*-test at *p* values < 0.01.

## Data Availability

The research article data used to support the findings of this study are included within the article.
